# Using Classical Test Theory to Determine the Psychometric Properties of the Italian Version of the Feeding/Swallowing Impact Survey

**DOI:** 10.3390/jcm14217607

**Published:** 2025-10-27

**Authors:** Valeria Crispiatico, Alessandra Baffi, Mariagrazia Anna Buratti, Lorenzo Montali, Renée Speyer

**Affiliations:** 1 Department of Psychology, University of Milano-Bicocca, Piazza dell’Ateneo Nuovo 1, 20126 Milan, Italy; v.crispiatico@campus.unimib.it (V.C.); lorenzo.montali@unimib.it (L.M.); 2 AIAS Busto Arsizio Onlus “Annibale Tosi”, Via Alba 30, 21052 Busto Arsizio, Italy; alessandra.baffilogo@gmail.com; 3 “Crescere Comunicando”, Via Carcano, 40, 21047 Saronno, Italy; crescerecomunicando@gmail.com; 4 School of Health Sciences, College of Medicine, Nursing & Health Sciences, University of Galway, H91 TK33 Galway, Ireland; 5 Curtin School of Allied Health, Curtin University, Perth, WA 6102, Australia

**Keywords:** FS-IS, psychometrics, reliability, validity, classical test theory, feeding disorders, quality of life, caregiver

## Abstract

**Background/Objectives:** The Italian version of the Feeding/Swallowing Impact Survey (FS-IS-IT) is an 18-item caregiver self-report questionnaire assessing the impact of paediatric feeding disorders (PFDs) on health-related quality of life (HR-QoL). The present study sought to evaluate its psychometric properties using Classical Test Theory (CTT), following COSMIN (COnsensus-based Standards for the selection of health Measurement INstruments) guidelines and criteria. **Methods:** A total of 145 caregivers of children with PFD of various etiologies were recruited (median age: 60.0 months; IQR: 35.8–108.0), of whom 134 provided sufficiently complete data for psychometric analysis. Structural validity was determined using exploratory factor analysis. Internal consistency was assessed using Cronbach’s alpha coefficient, McDonald’s ω, and inter-item correlations. Hypothesis testing was conducted using Mann–Whitney U-tests and correlation analysis, while interpretability was examined by assessing floor and ceiling effects. **Results:** Factor analysis indicated that the FS-IS-IT is a unidimensional measure, with an adequate total variance explained of 60.1%. The FS-IS-IT has moderate structural validity, good internal consistency with some evidence of item redundancy, strong construct validity as supported by hypothesis testing, and no floor and ceiling effects. **Conclusions:** These findings suggest that the FS-IS-IT is a promising caregiver self-report measure for evaluating HR-QoL in PFD. Further validation is recommended to assess potential item redundancy and to examine the dimensionality of the FS-IS-IT using item response theory. Conversely, although the Italian version of the FS-IS demonstrated encouraging psychometric properties, it could be further strengthened in future studies by revising ambiguous items, refining response formats, and removing misfitting items.

## 1. Introduction

### 1.1. Paediatric Feeding Disorder

A paediatric feeding disorder (PFD) is characterised by a disruption in oral nutrient intake that persists for at least two weeks and is not developmentally appropriate for the individual’s age [1]. This definition encompasses the ability to acquire and develop feeding skills, facilitating the transition from breastfeeding and/or bottle feeding to eating age-appropriate foods independently. The diagnostic framework for PFD comprises four equally significant domains: medical; nutritional; feeding skills; and psychosocial dysfunction [1]. PFD affects approximately one in 37 children under the age of five [2]. This multidisciplinary approach provides a comprehensive diagnostic framework for infants and young children with feeding difficulties arising from medical conditions, developmental concerns and/or skill-based challenges, which may subsequently lead to nutritional and/or psychosocial issues [1]. A PFD not only affects the child’s life but also significantly impacts the quality of life of caregivers, as caring for a child with swallowing and feeding difficulties has been shown to result in decreased overall well-being [1]. Numerous studies have documented elevated levels of parental anxiety and stress, social isolation, limitations in social activities and employment, as well as negative effects on family relationships [3,4,5]. Caregivers report various challenges, including time-related and financial burdens, along with concerns about choking risks and their potential repercussions for other family members [6,7]. Therefore, understanding the consequences of PFD is essential, as such knowledge is crucial for improving paediatric care, alleviating caregiver strain, and implementing appropriate interventions across diverse geographical and economic settings [8].

### 1.2. Psychometrics

It is also evident that self-reported measures play a crucial role in assessing the impact of PFD on caregivers’ lives, particularly in terms of health-related quality of life (HR-QoL), and should be considered an integral component of the comprehensive, multidimensional assessment of PFD. However, a measure is only appropriate for clinical or research use when it shows strong performance across all psychometric domains [9]. The COnsensus-based Standards for the selection of health Measurement INstruments (COSMIN) group established an international, consensus-driven taxonomy, terminology, and definitions related to the measurement properties of outcome measures [10]. The COSMIN framework [10] delineates nine psychometric properties across three domains: (1) validity, which refers to the extent to which an instrument accurately measures the construct it is intended to assess; (2) reliability, indicating the degree to which an instrument is free from measurement error; and (3) responsiveness, defined as the ability of an instrument to detect meaningful change over time in the construct being measured. Although interpretability—the degree to which clinical meaning can be assigned to an instrument’s quantitative scores or changes in scores—is not considered a psychometric property, it is clinically important. In addition, the COSMIN framework provides recommended statistical and methodological approaches along with detailed criteria to evaluate measurement properties. To select the most appropriate measures from available caregiver self-report questionnaires, it is essential to first evaluate and compare the psychometric properties of each instrument.

### 1.3. Feeding/Swallowing Impact Survey

In order to ascertain the extent of the impact on HR-QoL to feeding, Lefton-Greif and colleagues developed the Feeding/Swallowing Impact Survey (FS-IS) [11]. The FS-IS is an 18-item questionnaire divided into three subscales: (1) the “Daily Activity” subscale assesses the impact of managing swallowing and feeding difficulties on a daily basis (5 items); (2) the “Worries” subscale evaluates the caregivers’ concerns for the child’s health and well-being (7 items); and (3) the “Feeding Difficulties” subscale assesses the challenges associated with managing eating or swallowing difficulties (6 items). The questionnaire utilises a five-point Likert scale, ranging from 1 = “never” to 5 = “almost always”, with a “not applicable” (N/A) option included for each item [11]. Higher scores indicate a greater impact on the caregiver’s HR-QoL due to the child’s feeding or swallowing problems affecting their daily functioning. The total score and the score for each subscale are calculated by summing the item scores and dividing by the number of items answered, yielding an average score.

The original English version of the instrument [11] has been translated into several languages, including Turkish [12], Persian [13], and Brazilian Portuguese [14]. Although the initial development focused on infants with a mean age of approximately 14 months (interquartile range [IQR] 7–35 months) and included participants with diverse pathologies, subsequent cross-cultural validation studies have extended its use to older children [12,14] and a wide range of patient populations, including cases of eosinophilic esophagitis [15] esophageal atresia [16], Down syndrome [17], laryngeal cleft [18], and cleft palate [19]. Additionally, in some studies [14,20], the response option “Not Applicable” was removed.

Recently, the questionnaire has been cross-culturally adapted into Italian (FS-IS-IT) [21]. The final version of the FS-IS-IT was developed by incorporating feedback from a panel of clinical dysphagia experts to ensure its relevance, comprehensiveness and comprehensibility (i.e., content validity) [10]. Similarly to the original English questionnaire, the Italian version comprises 18 items, utilizing the same scoring system as the original FS-IS—a 5-point Likert scale with an option for “not applicable” for each item. After development, the FS-IS-IT was subsequently pretested with a group of Italian caregivers. This early cross-cultural adaptation study [21] provided preliminary psychometric data on the Italian FS-IS, including content validity and preliminary data on internal consistency, repeated measurement and convergent validity. Moreover, the study made an innovative comparison of scores between parental pairs (mother and father) of children with PFD (*n* = 32) and those of children with developmental disorders without PFD (*n* = 15). The analysis yielded findings indicating that the adverse impact on HR-QoL was more pronounced in parents of children with PFD than in parents of children without. Also, the study reported good diagnostic performance to differentiate between dyads of children with PFD and dyads without these symptoms. Furthermore, mothers and fathers of children with PFD experienced comparable levels of impact on HR-QoL. However, due to the limited sample size, the reported psychometric data can only be considered pilot data, and it was not possible to perform a factor analysis to confirm the questionnaire’s structural validity. Consequently, only preliminary conclusions can be drawn from this investigation.

### 1.4. Aim of Study

The aim of this cross-sectional study was to verify the psychometric properties of the Italian version of the FS-IS using Classical Test Theory (CTT) in accordance with the COSMIN framework and methodological guidelines, thereby aligning with current psychometric standards. Establishing robust psychometric properties across these domains is essential to justify its application in clinical practice and research. Specifically, this study aimed to determine internal consistency, structural validity, hypothesis testing for construct validity and interpretability.

## 2. Methods

### 2.1. Study Design

This prospective, cross-sectional study was conducted in accordance with the Declaration of Helsinki. It was previously approved by [Masked].

### 2.2. Participant Recruitment

Parents of children with PFD were enrolled by convenience sampling at [Masked] between September 2024 and May 2025. Participation in the study was voluntary, with parents retaining the right to withdraw at any time, and written informed consent was obtained from all participants prior to participation in accordance with ethical standards.

The inclusion criteria were: (1) the child must have a diagnosis of a neurological condition (e.g., cerebral palsy, intellectual disability, developmental delay), a congenital condition (e.g., tracheoesophageal fistula), an acquired condition (e.g., tracheostomy, vocal fold paralysis or paresis), or a genetic/chromosomal anomaly (e.g., Down syndrome, other syndromes or congenital metabolic disorders); (2) the child must present with feeding or swallowing problems, as determined through a clinical examination conducted by an experienced speech and language pathologist (SLP); (3) participants were required to have adequate ability to read or understand spoken Italian in order to understand the purpose of the research; (4) parents were required to be over 18 years of age.

### 2.3. Protocol

The data collection process was overseen by an experienced speech-language pathologist (SLP). The caregiver was tasked with the completion of an online form, the purpose of which was to gather information pertaining to both the sociodemographic and demographic characteristics of the child. In addition to this, the form also requested details about the child’s history with regard to feeding and swallowing (for example, the number of hospital admissions, the time at which difficulties with food and liquids first became apparent, and any signs of a feeding or swallowing disorder). Subsequently, during the onsite visit, the caregiver completed the electronic version of the FS–IS–IT in presence of the SLP [21]. Furthermore, the SLP classified the children’s diet according to the IDDSI Functional Diet Scale [22] based on mealtime descriptions. This scale was developed using a matrix—similar to a mileage chart—that serves as an adjunct to the IDDSI framework [23]. The matrix is a practical tool that is used to assess the degree of dietary texture restriction recommended for a patient. The scale ranges from 0 (no oral intake) to 8 (no dietary restriction), with lower scores indicating more stringent dietary texture restrictions. The IDDSI Functional Diet Scale was selected as an outcome measure because it provides a standardized, cross-culturally validated classification of dietary textures, supporting both clinical practice and research, while broadly reflecting rheological properties (e.g., [24,25]).

### 2.4. Statistical Analysis

The measurement properties of the Italian version of FS-IS (FS-IS-IT) were determined according to the COSMIN taxonomy of psychometric properties and definitions for health-related outcomes [26,27]. The collected data were used to evaluate various psychometric properties, namely structural validity, internal consistency and hypothesis testing for construct validity, as well as interpretability. Although interpretability is not classified as a psychometric property, it is an important attribute of a measure, allowing quantitative scores or changes in scores to be translated into meaningful qualitative information [28].

The expected range of missing data within any given dataset is between 4% and 10% [29]. For the purposes of statistical analysis, N/A responses were treated as missing values, consistent with the approach adopted by Lefton-Greif et al. [11]. Depending on the extent of missing data (i.e., incomplete datasets and N/A responses), participants may have been excluded from specific analyses to ensure that the proportion of missing data per item remained below the 10% threshold. The decision to use parametric analyses (for normally distributed data) or nonparametric statistical analyses (for non-normally distributed data) was based on the normality of the data. A *p*-value less than 0.05 was considered to be statistically significant. Statistical procedures were performed using IBM SPSS Statistics 26.0^®^ software package for Mac (SPSS Inc., Chicago, IL, USA).

***Structural validity***. Structural validity is the degree to which scores reflect the dimensionality of the construct to be measured. Assessing structural validity requires critical evaluation of the dataset’s suitability for factor analysis, including sample size adequacy, as determined by the Kaiser-Meyer-Olkin (KMO) test and Bartlett’s Test of Sphericity [30]. The Kaiser-Meyer-Olkin (KMO) measure ranges from 0.0 to 1.0, while Bartlett’s Test of Sphericity yields a significance value (*p*-value). A KMO value greater than 0.6 and Bartlett’s test result with *p* < 0.05 indicate that the dataset is sufficiently adequate for factor analysis [30].

In accordance with COSMIN guidelines, a very good sample size was defined as at least seven participants per item and a total sample of ≥100 [31]. To evaluate the underlying structure of the instrument, exploratory principal component factor analysis with Promax rotation was conducted, followed, where appropriate, by confirmatory factor analysis using the maximum likelihood method.

***Internal consistency***. Internal consistency is the degree of interrelatedness among items. It was examined by calculating Cronbach’s alpha and McDonald’s ω for the whole questionnaire and for each subscale where applicable, as well as by analysing inter-item correlations. A Cronbach’s alpha below 0.70 indicates inadequate internal consistency, while a value above 0.90 may suggest item redundancy [32].

***Hypothesis testing for construct validity***. Hypothesis testing for construct validity is defined as the extent to which the scores obtained from a given measure align with the established hypotheses. These hypotheses may concern internal relationships, correlations with other measures, or differences between relevant groups. This approach rests on the assumption that the measure accurately represents the construct being studied [28]. The following hypotheses were tested: (1) the impact of caregiver-related feeding/swallowing disorders on HR-QoL is not expected to differ between male and female caregivers; therefore, the FS-IS-IT total score is hypothesised to be independent of the caregiver’s gender (independent samples t-test or Mann–Whitney *U* test); (2) the FS-IS-IT total score is not expected to differ based on the gender of the child (independent samples t-test or Mann–Whitney *U* test); (3) the FS-IS-IT total score is expected to be associated with the IDDSI Functional Diet Scale score (Pearson or Spearman correlation); and (4) the age of the children is not expected to be associated with the FS-IS-IT total score (Pearson or Spearman correlation).

***Interpretability***. Interpretability was assessed by examining the FS-IS-IT for floor and ceiling effects, defined as occurring when more than 15% of participants achieve the lowest or highest possible score, respectively [27]. The presence of such effects indicates a lack of items targeting the extremes of the scale, which may compromise both the content validity and reliability of the measure [31].

## 3. Results

### 3.1. Participants and Sample Characteristics

A total of 145 children and their caregivers were included in this study. Although there were no missing responses due to incomplete FS-IS-IT surveys, several participants selected the “not applicable” (N/A) response option, as reported in the original study by Lefton-Greif et al. [11]. In line with the methodology of Lefton-Greif et al. [11], N/A responses were treated as missing values for statistical analysis. Of the 145 participants, 36 caregivers used the N/A response option for one or more items, and 11 of these used the N/A response more than twice. Participants with more than two missing values were excluded from statistical analyses, resulting in a final sample size of 134 children and their caregivers.

Caregivers (*n* = 134) were aged 25–66 years, with a median age of 40.2 years (IQR 34.0–45.0), while children ranged in age from 2 to 164 months, with a median age of 60.0 months (IQR: 35.8–108.0). Children presented with a broad variety of diagnoses: congenital and genetic syndromes (*n* = 33; 24.6%), structural and craniofacial syndromes (*n* = 28; 20.9%), neurological and neurodevelopmental syndromes (*n* = 24; 17.9%), cerebral palsy (*n* = 18; 13.4%), metabolic and endocrine syndromes (*n* = 13; 9.7%), muscular dystrophies (*n* = 12; 9.0%), and other diseases or disorders such as esophageal problems or prematurity (*n* = 6; 4.5%). Table 1 presents the demographic and clinical characteristics of the included children and their caregivers.

### 3.2. Structural Validity

Only participants with less than three N/A responses were included (*n* = 134) to ensure that the proportion of missing data per item remained below the 10% threshold [29]. This sample was deemed adequate for factor analysis, meeting the minimum requirement according to COSMIN of 7 subjects per item (7 × 18 = 126) and a total sample size of ≥100 [31]. The suitability for the dataset for factor analysis was confirmed with Kaiser-Meyer-Olkin (KMO) indexes of 0.899, and Bartlett’s Test of Sphericity indicated values of *p* < 0.001 [30].

An exploratory Principal Component factor analysis with Promax rotation, accounting for the expected inter-item correlations, was performed on all FS-IS-IT items. The analysis identified three factors that together explained 60.10% of the total variance. This level of explained variance is deemed adequate, as recommended thresholds typically range from 50% [33] to 60% [34]. Notably, the majority of the items loaded on the first factor, which accounted for 46.52% of the total variance, while the second and third factors explained 7.78% and 5.80%, respectively. Consequently, unidimensionality can be assumed based on two criteria proposed by Prinsen et al. [10]: (1) the first factor accounts for at least 20% of the variability; and (2) the ratio of the variance explained by the first factor to the second factor (σ^2^_Factor 1_/σ^2^_Factor 2_) exceeds the threshold of 4. It can thus be concluded that the ratio of the variance explained by the first factor to that of the second factor is greater than 4 (46.52/7.78 = 5.94). Therefore, in accordance with the established guidelines [10], this analysis yielded a unidimensional factor.

Confirmatory factor analysis based on a three-factor model (representing the three underlying subscales of the original FS-IS) was not conducted due to the unidimensional nature of the Italian version of the FS-IS scale. Since the results of the factor analyses strongly suggest that the FS-IS-IT represents a unidimensional construct, it was determined that subsequent analyses would focus solely on the FS-IS-IT total score.

### 3.3. Internal Consistency

The data were found to be non-normally distributed (*n* = 134); therefore, non-parametric Spearman’s rank-order correlation coefficients were calculated. Table 2 presents the heat map of the absolute values of the correlation matrix including all FS-IS-IT items (0.10 ≤ *r*_s_ ≤ 0.67). The determinant value of 3.5000 × 10^−5^ exceeds the conventional cutoff of 0.00001, indicating acceptable levels of inter-item correlation and confirming suitability for factor analysis [35]. No multicollinearity was present as all correlations were ≤0.669, remaining below the commonly accepted threshold of 0.80 [35,36]. The strength of *r*-values can be interpreted based on Cohen’s classification: 0.10 as weak, 0.30 as moderate, and 0.50 as strong in terms of magnitude [37]. The mean correlation for items within the FS-IS-IT total score was moderate to strong (*r*_s_ = 0.420; SD = 0.103). Cronbach’s alpha and McDonald’s ω were calculated for the total FS-IS-T, yielding values of 0.93 and 0.93, respectively. These results indicate adequate internal consistency, while also suggesting the potential for item redundancy.

### 3.4. Hypothesis Testing for Construct Validity

PlMissing data were handled using listwise deletion (*n* = 109), after which non-parametric statistical methods were employed due to the non-normal distribution of the data. Hypothesis testing for construct validity was assessed for the following four hypotheses:

(1) The hypothesis that there would be no significant difference in the impact of caregiver-related feeding and swallowing disorders on health-related quality of life (HR-QoL) between genders was supported by the Mann–Whitney *U* test. No significant differences between male (*n* = 33) and female caregivers (*n* = 76) were identified on the FS-IS-IT total score: Mean Rank_Male_ = 50.79 (Sum of Ranks = 1676.00); Mean Rank_Female_ = 56.83 (Sum of Ranks = 4319.00); *U* = 1115.000; *p* = 0.359, two-tailed.
(2) The hypothesis that the FS-IS-IT total score would not differ based on the gender of the child was confirmed, as no significant differences were found between boys (*n* = 51) and girls (*n* = 58): Mean Rank_Male_ = 54.58 (Sum of Ranks = 2783.50); Mean Rank_Female_ = 55.37 (Sum of Ranks = 3211.50); *U* = 1457.500; *p* = 0.896, two-tailed.
(3) The hypothesis that the FS-IS-IT total score would be associated with the IDDSI Functional Level score was supported (*r*_s_ = −0.502, *p* < 0.001).
(4) The hypothesis that the age of the children would not be associated with the FS-IS-IT total average score was confirmed (*r*_s_ = −0.183, *p* = 0.60).

### 3.5. Interpretability

**Figure 1:**
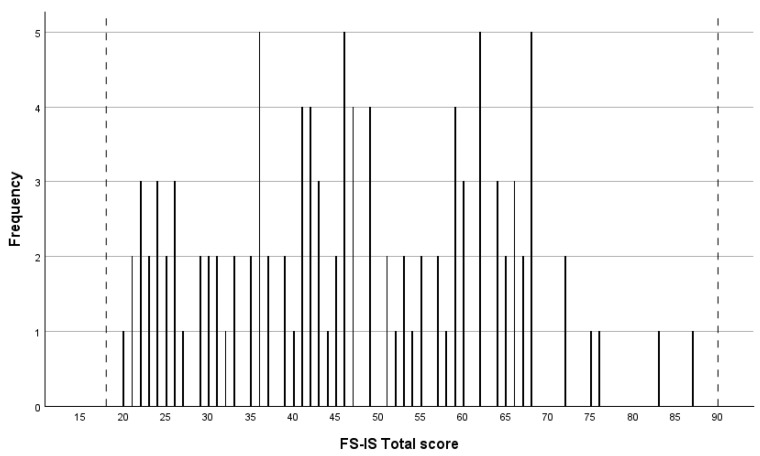
Data distribution for the FS-IS-IT total score. Note. The dotted lines represent the minimum and maximum scores attainable on the FS-IS-IT.

The means and standard deviations for the FS-IS-IT total score were determined (see Table 1). Floor and ceiling effects (Figure 1) were assessed using only complete datasets, with listwise deletion applied for any missing data (*n* = 109). The FS-IS-IT total score is a scale ranging from 18 to 90. Findings demonstrated that 0% of participants achieving either the lowest or highest possible score, suggesting that the FS-IS-IT total score exhibited no evidence of floor or ceiling effects.

## 4. Discussion

The present study investigates the measurement properties of the Italian translation of the FS-IS, a self-administered questionnaire for caregivers that assesses HR-QoL in caregivers of a child with PFD. The study employs CTT, a framework that evaluates the overall performance of a measure, with psychometric findings specific to the sample population used in the evaluation [38]. The standardised nomenclature and conceptualisations for the measurement characteristics of health-related patient-reported outcome measures provided by the COSMIN framework were utilised [26,31].

### 4.1. Reliability and Validity

The results of the factor analysis, together with the high Cronbach’s alpha and McDonald’s omega coefficient for the overall FS-IS-IT score—consistent with the preliminary analysis by Baffi et al. [21]—suggest that the Italian version is likely unidimensional, with some items potentially exhibiting redundancy. These findings indicate that all items appear to measure a single underlying construct. Consequently, interpretation of subscale scores is not recommended for the Italian version, as they do not accurately represent the unidimensional structure of the FS-IS-IT. In contrast to the English original questionnaire [11] and the Brazilian Portuguese adaptation [14], the Italian adaptation demonstrated that the items intended to reflect different PFD-related aspects of caregivers’ daily life—whether addressing the impact on daily activities (“Daily Activity”), psychosocial concerns (“Worry”), or functional challenges related to meal management (“Feeding Difficulties”)—were highly interrelated and could not be clearly distinguished. The different aspects as defined and represented by the 18 items of the FS-IS-IT, proved to be intertwined, making it difficult to divide them into distinct subscales. However, the percentage of explained variance indicates that the current FS-IS-IT items adequately capture the construct, thereby supporting the questionnaire’s overall efficacy in assessing the primary aspects of caregiver HR-QoL associated with PFD.

Hypothesis testing for construct validity confirmed all four hypotheses. The findings demonstrated a negative correlation between FS-IS-IT total scores and diet restriction, as measured by the Diet Functional Scale. Furthermore, there was no statistically significant difference in FS-IS-IT total scores based on the gender of the care provider. Consistent with the findings of Baffi et al. [21], the degree of impact on HR-QoL was comparable across caregiver genders. As hypothesised, no statistically significant correlations were found between the child’s gender or age and FS-IS-IT total scores. Based on histogram analysis the FS-IS-IT demonstrated no floor and ceiling effects, suggesting that the scale comprehensively captures the full spectrum of caregiver responses, with no items missing at the lower or upper ends.

In contrast to Baffi et al. (2025) [21], this study used a sufficiently large sample to perform factor analysis and to evaluate the psychometric properties of the Italian version of the FS-IS through a comprehensive validation approach [26,27,31]. The findings of the present study are incongruent with those reported by Lefton-Greif et al. [11] and Rama et al. [14], who identified a three-factor structure in the English and Brazilian Portuguese versions of the questionnaire. However, it should be noted that the sample size in the study of Rama et al. [14] (*n* = 90) was smaller than that of the present study and insufficient for evaluating structural validity according to COSMIN guidelines [26,27].

In addition, during the Italian data collection, several participants reported confusion about how to interpret certain questions. This confusion appeared to stem not from translation or language issues, but from ambiguity in the formulation of specific items, suggesting the need for clearer wording or additional instructions in future adaptations. For example, parents of children with gastrostomies found it difficult to answer item 13 (‘It is difficult to feed my child because it takes a long time to prepare food and drinks in the “right” way’) and item 14 (It is difficult to feed my child because I don’t know how to prepare food and drinks), and were unsure whether to select ‘never’ or ‘N/A’. This feedback highlights potential issues with item clarity that may have influenced response accuracy. Additionally, the study by Lefton-Greif et al. [11] did not provide clear guidance on how to handle the “N/A” response option in data analysis, which may impact consistency in scoring and interpretation.

The validation of the IT-FSIS has important theoretical and practical implications. Its unidimensional structure indicates that caregiver burden in pediatric feeding disorders is an integrated construct encompassing daily activities, psychosocial well-being, and feeding challenges. The validated Italian version offers a preliminary, culturally adapted measure for clinicians and researchers to assess caregiver HR-QoL, guide support, monitor outcomes, and inform future refinements to improve clarity and usability.

### 4.2. Future Research

These findings suggest that the FS-IS-IT is a promising caregiver self-report measure for evaluating health-related quality of life (HR-QoL) in children with PFD. However, this study employed CTT as a framework to assess the measure’s overall performance, yielding psychometric findings specific to the sample population [38,39]. Unlike CTT, the more recently developed Item Response Theory (IRT) approach focuses on individual items as the unit of analysis and produces results that are independent of the specific sample characteristics. Given the limitations of CTT, future studies with larger samples should consider applying IRT, which is widely regarded as the leading framework for developing measures and evaluating psychometric properties [38,39]. While CTT procedures and interpretations are relatively straightforward, IRT offers the advantage of assessing the reliability and performance of each item and its contribution to the overall construct.

## 5. Limitations and Future Research

The absence of repeated measurements in this study precluded the assessment of measurement error and test–retest reliability. Additionally, responsiveness was beyond the scope of this study and warrants examination in future research. This study relied exclusively on CTT for the psychometric evaluation of the FS-IS-IT. While CTT provides a foundational approach for validation, future research should incorporate CTT alongside IRT to strengthen the psychometric evidence of the FS-IS-IT [28,40]. This combined approach would offer a more comprehensive understanding and strengthen the existing psychometric evidence for the FS-IS-IT.

## 6. Conclusions

CTT analyses indicate that the FS-IS-IT is a promising self-report measure of HR-QoL for caregivers of children with PFD, with evidence of a unidimensional structure. The measure demonstrates moderate structural validity, good internal consistency, and strong construct validity as supported by hypothesis testing. Further research is recommended to assess potential item redundancy and to investigate the dimensionality of the FS-IS-IT using item response theory. On the other hand, although the Italian version of the FS-IS also showed encouraging psychometric properties, future studies may consider strengthening the questionnaire by revising ambiguous items, modifying response options, and removing misfitting items.

## Figures and Tables

**Table 1:** Demographic and clinical characteristics of study population (*n* = 134).

Variable	*n* (%); Median (IQR) *; Range
***Gender carer***	
Male	42 (31.3%)
Female	92 (68.7%)
***Age Carer*** (years)	Median 40.2 (IQR 34.0–45.0) Range: 25–66
***Work Activity***	
Full-time	79 (59.0%)
Part-time	30 (22.4%)
Not working	25 (18.6%)
***Educational Level***	
Secondary Education or lower	67 (50.0%)
Tertiary Education	67 (50.0%)
***Child Gender***	
Male	66 (49.3%)
Female	68 (50.7%)
***Child Age*** (months)	Median 60.0 (IQR 35.8–108.0) Range: 2–164
***Diagnosis***	
Congenital and genetic syndromes	33 (24.6%)
Structural and craniofacial syndromes	28 (20.9%)
Neurological and neurodevelopmental syndromes	24 (17.9%)
Cerebral Palsy	18 (13.4%)
Metabolic and endocrine syndromes	13 (9.7%)
Muscular Dystrophies	12 (9.0%)
Other diseases or disorders	6 (4.5%)
***IDDSI Functional Diet Scale*** (IDDSI-FDS)	Median 5.7 (IQR 5.0–7.0) Range: 0–8
***FS-IS Total Score*** **	Median 46.9 (IQR 35.0–60.0) Range: 20–87

Note. * Interquartile range; ** *n* = 109.

**Table 2:** Heat map of the absolute values of the correlation matrix including all FS-IS-IT items (*n* = 134).

	1	2	3	4	5	6	7	8	9	10	11	12	13	14	15	16	17	18	
**1**	1.000	0.578	0.546	0.516	0.638	0.417	0.440	0.437	0.448	0.439	0.395	0.587	0.611	0.324	0.241	0.481	0.452	0.508	**1**
***n***	130	126	125	129	128	129	130	128	127	124	129	126	127	126	119	120	123	126	***n***
**2**	-	1.000	0.669	0.388	0.481	0.399	0.561	0.452	0.508	0.445	0.447	0.503	0.453	0.344	0.148	0.377	0.526	0.507	**2**
***n***	**-**	129	127	129	128	128	129	127	126	123	128	125	126	125	118	118	122	125	***n***
**3**	-	-	1.000	0.486	0.507	0.443	0.413	0.451	0.449	0.459	0.384	0.586	0.373	0.227	0.281	0.350	0.476	0.442	**3**
***n***	-	-	129	128	126	128	129	127	125	123	128	125	126	125	118	118	122	124	***n***
**4**	-	-	-	1.000	0.557	0.320	0.356	0.380	0.373	0.475	0.518	0.519	0.427	0.318	0.130	0.452	0.422	0.313	**4**
***n***	**-**	-	-	133	131	132	133	131	129	126	132	129	130	129	122	121	126	128	***n***
**5**	-	-	-	-	1.000	0.491	0.534	0.419	0.392	0.446	0.479	0.573	0.571	0.385	0.214	0.437	0.429	0.490	**5**
***n***	-	-	-	-	131	130	131	129	128	125	130	127	128	127	120	120	124	127	***n***
**6**	-	-	-	-	-	1.000	0.511	0.379	0.342	0.515	0.465	0.328	0.472	0.298	0.257	0.453	0.419	0.455	**6**
***n***	-	-	-	-	-	133	133	131	130	126	132	129	130	129	122	121	126	129	***n***
**7**	-	-	-	-	-	-	1.000	0.197	0.358	0.173	0.461	0.524	0.295	0.383	0.127	0.345	0.323	0.266	**7**
***n***	-	-	-	-	-	-	134	132	130	127	133	130	131	130	123	122	127	129	***n***
**8**	-	-	-	-	-	-	-	1.000	0.079	0.268	0.247	0.210	0.210	0.221	0.060	0.186	0.224	0.256	**8**
***n***	-	-	-	-	-	-	-	132	129	127	131	129	130	129	122	121	126	128	***n***
**9**	-	-	-	-	-	-	-	-	1.000	0.083	0.283	0.255	0.157	0.232	0.130	0.195	0.170	0.091	**9**
***n***	-	-	-	-	-	-	-	-	130	126	130	127	128	127	120	120	124	128	***n***
**10**	-	-	-	-	-	-	-	-	-	1.000	0.230	0.177	0.264	0.291	0.269	0.368	0.326	0.402	**10**
***n***	-	-	-	-	-	-	-	-	-	127	127	125	126	125	118	118	122	124	***n***
**11**	-	-	-	-	-	-	-	-	-	-	1.000	0.730	0.465	0.515	0.060	0.375	0.383	0.372	**11**
***n***	-	-	-	-	-	-	-	-	-	-	133	130	131	130	123	122	126	128	***n***
**12**	-	-	-	-	-	-	-	-	-	-	-	1.000	0.504	0.491	0.141	0.407	0.363	0.296	**12**
***n***	-	-	-	-	-	-	-	-	-	-	-	130	129	128	122	120	124	126	***n***
**13**	-	-	-	-	-	-	-	-	-	-	-	-	1.000	0.492	0.111	0.450	0.389	0.332	**13**
***n***	-	-	-	-	-	-	-	-	-	-	-	-	131	129	123	121	124	127	***n***
**14**	-	-	-	-	-	-	-	-	-	-	-	-	-	1.000	0.255	0.523	0.609	0.508	**14**
***n***	-	-	-	-	-	-	-	-	-	-	-	-	-	130	123	121	124	125	***n***
**15**	-	-	-	-	-	-	-	-	-	-	-	-	-	-	1.000	0.357	0.270	0.279	**15**
***n***	-	-	-	-	-	-	-	-	-	-	-	-	-	-	123	115	119	119	***n***
**16**	-	-	-	-	-	-	-	-	-	-	-	-	-	-	-	1.000	0.535	0.460	**16**
***n***	-	-	-	-	-	-	-	-	-	-	-	-	-	-	-	122	117	118	***n***
**17**	-	-	-	-	-	-	-	-	-	-	-	-	-	-	-	-	1.000	0.484	**17**
***n***	-	-	-	-	-	-	-	-	-	-	-	-	-	-	-	-	-	124	***n***
**18**	-	-	-	-	-	-	-	-	-	-	-	-	-	-	-	-	-	1.000	**18**
***n***	-	-	-	-	-	-	-	-	-	-	-	-	-	-	-	-	-	129	***n***
	**1**	**2**	**3**	**4**	**5**	**6**	**7**	**8**	**9**	**10**	**11**	**12**	**13**	**14**	**15**	**16**	**17**	**18**	
*Legend:*																			
									0 ≤ *r* < 0.2										
									0.2 < *r* < 0.4										
									0.4 < *r* < 0.6										
									0.6 < *r* < 0.8										
									0.8 < *r* ≤ 1										

Note. Determinant = 3.5000 × 10^−5^.

## Data Availability

The datasets generated during and analyzed during the current study are available from the corresponding author on reasonable request.
